# Postpartum acquired hemophilia A: immunopathogenesis, diagnostic challenges, and treatment with the CyDRi protocol

**DOI:** 10.3389/pore.2026.1612302

**Published:** 2026-05-04

**Authors:** Ágnes Szappanos, Hajnalka Andrikovics, Péter Varga, Mónika Fekete, Vince Fazekas-Pongor, Márk Lehoczki, Viktória Király, Beáta Vilimi, Gábor Mikala, Imre Bodó, Andrea Ceglédi

**Affiliations:** 1 Heart and Vascular Center, Semmelweis University, Budapest, Hungary; 2 Department of Rheumatology and Clinical Immunology, Semmelweis University, Budapest, Hungary; 3 Fodor Center for Prevention and Healthy Aging, Semmelweis University, Budapest, Hungary; 4 Laboratory of Molecular Genetics, Central Hospital of Southern Pest, National Institute for Hematology and Infectious Diseases, Budapest, Hungary; 5 Institute of Preventive Medicine and Public Health, Semmelweis University, Budapest, Hungary; 6 Doctoral College, Health Sciences Division, Semmelweis University, Budapest, Hungary; 7 Faculty of Physical Therapy, University of Pécs, Pécs, Hungary; 8 Central Laboratory of Central Hospital of Southern Pest, National Institute of Hematology and Infectious Diseases, Budapest, Hungary; 9 Department of Internal Medicine and Hematology, Semmelweis University, Budapest, Hungary; 10 Department of Hematology and Medical Oncology, Emory University, Atlanta, GA, United States; 11 Departments of Hematology and Stem Cell Transplantation, South Pest Central Hospital, National Institute of Hematology and Infectious Diseases, Budapest, Hungary

**Keywords:** autoimmunity, case illustration, CyDRi protocol, FVIII inhibitor, immunosuppression

## Abstract

Acquired hemophilia A (AHA) is a rare, potentially life-threatening autoimmune bleeding disorder characterized by the development of inhibitory autoantibodies against factor VIII (FVIII). While most commonly diagnosed in the elderly, AHA can also occur in the postpartum period, where it presents unique diagnostic and therapeutic challenges. This review provides a comprehensive overview of the pathogenesis, clinical features, diagnostic approaches, and current therapeutic strategies for postpartum AHA. We highlight the immunological shifts during pregnancy and the postpartum period that may contribute to the breakdown of immune tolerance and the emergence of FVIII autoantibodies. Key aspects of laboratory diagnosis are outlined, including the role of coagulation screening, mixing studies, and inhibitor assays. We compare the efficacy and safety of established immunosuppressive regimens, with a particular focus on the CyDRi protocol—a combination of cyclophosphamide, dexamethasone, and rituximab—which has demonstrated high rates of complete remission with a favorable toxicity profile. To illustrate clinical application, we describe a case of severe postpartum AHA managed successfully with the CyDRi protocol, followed by an uneventful subsequent pregnancy. With timely diagnosis and appropriately tailored immunosuppressive therapy, postpartum AHA can be effectively treated, and favorable hematologic and reproductive outcomes are achievable.

## Introduction

Acquired hemophilia A (AHA) is a rare but potentially life-threatening autoimmune coagulopathy characterized by the development of autoantibodies—most commonly of the IgG1 and IgG4 subclasses—against coagulation factor VIII (FVIII). These neutralizing antibodies disrupt the intrinsic coagulation pathway, leading to spontaneous, often severe bleeding episodes involving soft tissues, mucous membranes, and muscles, without any preceding trauma or injury [1–5]. The incidence of AHA is estimated at 1 to 1.78 cases per million individuals annually [1, 5–7]. Unlike congenital hemophilia, AHA typically affects individuals with no prior personal or family history of bleeding, posing diagnostic challenges and delaying treatment in many cases.

Among the various secondary forms of AHA, the postpartum setting represents a distinctive and underrecognized subset. Postpartum AHA typically emerges within 1–4 months after delivery and accounts for approximately 2%–15% of all reported AHA cases [8–25]. Although rare, its clinical relevance is considerable, particularly due to the risk of life-threatening hemorrhage in young, otherwise healthy women and the implications for future reproductive health. The disease typically is often manifesting as deep soft tissue bleeding, muscle hematomas, or persistent vaginal hemorrhage [10, 11, 13–22, 25]. Diagnosis is often delayed, as bleeding is frequently attributed to obstetric complications, such as uterine atony or birth trauma, and clinicians may not immediately consider acquired coagulopathy in the differential diagnosis.

While the general pathophysiology and management of AHA are increasingly understood, evidence regarding postpartum AHA remains limited to case reports, registry data, and small case series [8–25]. Moreover, very few reports provide long-term follow-up data on fertility or pregnancy outcomes after remission, leaving a gap in clinical guidance for managing affected women.

This narrative review aims to synthesize current knowledge on the immunopathogenesis, diagnosis, and treatment of postpartum AHA, with particular emphasis on the use of the CyDRi protocol—an innovative and well-tolerated immunosuppressive strategy. To ground this discussion in real-world clinical practice, we present a detailed illustrative case of postpartum AHA successfully treated with the CyDRi protocol. Importantly, we also report the patient’s subsequent uneventful pregnancy and delivery, offering new insights into the safety and feasibility of future childbearing following recovery from AHA.

## Immunopathogenesis of postpartum AHA

The immunological basis of postpartum AHA is complex and incompletely understood, but accumulating evidence suggests that it arises from a dysregulated immune rebound following pregnancy. During gestation, the maternal immune system is carefully modulated to tolerate the semi-allogeneic fetus. This adaptation is characterized by a shift from a Th1- to a Th2-dominant milieu, favoring anti-inflammatory responses and enhanced regulatory T-cell (Treg) activity. High levels of estrogen and progesterone promote the expansion of Tregs and the secretion of Th2 cytokines such as IL-4 and IL-10, which suppress cytotoxic and pro-inflammatory Th1 and Th17 pathways, thereby maintaining fetal tolerance [26, 27].

This finely balanced state, however, is abruptly reversed after delivery. The sudden decline in pregnancy-associated hormones, particularly estrogen and progesterone, is accompanied by a rapid contraction of Treg populations and a reactivation of Th1 and Th17 pathways. This postpartum “immune rebound” is associated with increased production of pro-inflammatory cytokines, heightened cytotoxic T-cell activity, and loss of tolerance to self-antigens, especially in genetically predisposed women [28, 29]. In parallel, innate immune cells such as decidual macrophages, dendritic cells, and natural killer (NK) cells become activated in the early postpartum period, with enhanced Toll-like receptor signaling that can prime autoreactive adaptive responses [30]. The convergence of these processes creates an environment in which autoreactive B cells can become activated and produce pathogenic IgG autoantibodies, predominantly of the IgG1 and IgG4 subclasses. In AHA, these antibodies specifically target domains of FVIII, neutralizing its procoagulant function and precipitating severe bleeding [2].

Although the immunological sequence leading to postpartum AHA is increasingly understood, the genetic factors conferring susceptibility remain an area of active investigation. Certain HLA class I and II alleles, including *A*03:01*, *DRB1*13:03*, *DRB1*16*, and *DQB1*05:02*, have been associated with heightened risk [31]. In addition, variants in key immune regulatory genes—such as the CTLA4 49 A/G polymorphism—and FVIII gene polymorphisms (c.8899G>A, c.3951C>G, c.6238G>A) may alter immune recognition thresholds. Emerging data also implicate NK-cell–related genes, such as *KLRK1* (rs1049174), in modulating susceptibility to immune dysregulation and inhibitor development. Thus, postpartum AHA likely represents the end result of a multifactorial process in which abrupt hormonal withdrawal, immune reactivation, loss of regulatory control, and predisposing immunogenetic factors converge to trigger the production of anti-FVIII autoantibodies. This mechanistic model explains why the disorder often emerges in previously healthy women after otherwise uncomplicated pregnancies and highlights potential avenues for risk stratification and prevention.

In sum, postpartum AHA appears to arise from a perfect storm of hormonal, cellular, and genetic factors converging during the delicate period of immune recalibration after delivery. While the condition is often monophasic and self-limited, its acute presentation can be severe, and understanding these immunological mechanisms is essential for guiding both diagnosis and therapeutic decision-making.

## Clinical manifestation and diagnosis of postpartum AHA

A hallmark clinical clue is the disproportion between the severity of bleeding and the apparent inciting trauma. The diagnosis of AHA requires a high index of suspicion and is established based on three core laboratory features:

1. Isolated prolonged activated partial thromboplastin time (aPTT) with a normal prothrombin time (PT), indicating a defect in the intrinsic coagulation pathway.
2. Failure of the mixing study to correct the aPTT, even after 2-h incubation, which suggests the presence of an inhibitor rather than a factor deficiency.
3. Markedly reduced FVIII activity, usually below 5%, coupled with a positive FVIII inhibitor titer as measured by the Bethesda assay.

In postpartum women, distinguishing AHA from obstetric hemorrhage can be particularly challenging; therefore, early inclusion of coagulation studies and mixing tests is essential when bleeding is disproportionate or unresponsive to standard obstetric interventions.

## Management and therapeutic approaches

The treatment of AHA, including postpartum cases, follows a dual-pronged approach [1]: achieving hemostatic control in the acute phase, and [2] eradicating the FVIII inhibitor with immunosuppressive therapy [2]. Timely and effective management is critical, as bleeding episodes may be life-threatening and can rapidly evolve into severe complications such as compartment syndrome or hemoperitoneum.

### Hemostatic therapy

The immediate clinical goal in AHA is to arrest bleeding. Acute bleeding in AHA is often unresponsive to FVIII replacement due to the presence of neutralizing autoantibodies. As such, first-line hemostatic therapy relies on bypassing agents, primarily recombinant activated factor VII (rFVIIa) or activated prothrombin complex concentrate (aPCC). The choice between these agents depends on availability, institutional preference, and thrombotic risk profile.

Recently, emicizumab, a bispecific monoclonal antibody that mimics the function of FVIII by bridging activated factor IX and factor X, has emerged as a promising therapeutic alternative in AHA [32–34]. Though originally developed and approved for congenital hemophilia A with or without inhibitors, off-label use of emicizumab in acquired hemophilia has been reported in several case series and observational studies, with encouraging results [32, 33]. Its favorable safety profile, subcutaneous administration, and prolonged half-life make it an attractive option for bleeding prophylaxis or adjunctive therapy, particularly in patients at high risk for recurrent hemorrhage. However, its role in acute bleeding management remains limited, as its onset of action is not immediate. While not yet incorporated into standard guidelines for AHA, emicizumab may represent an important component of future treatment algorithms. Once hemostasis is achieved, therapeutic focus must shift toward inhibitor eradication through timely and effective immunosuppression [4, 35–37].

### Inhibitor eradication with immunosuppressive therapy

Once hemostasis is achieved or underway, the priority shifts to eliminating the inhibitor, thereby reducing long-term bleeding risk and permitting normalization of FVIII levels. Standard immunosuppressive regimens include corticosteroids, either alone or in combination with cytotoxic agents (e.g., cyclophosphamide), or monoclonal antibodies (e.g., rituximab). Historically, a stepwise escalation approach has been favored, starting with steroids and adding other agents upon non-response. However, this sequential model may prolong exposure to bleeding risk and increase the chance of disease refractoriness. To overcome these limitations, we developed and validated the CyDRi protocol, a combinatorial immunosuppressive regimen delivered in a pulse schedule [38]. The CyDRi protocol consists of:

- Cyclophosphamide 1000 mg IV on days 1 and 22,
- Dexamethasone 40 mg IV on days 1, 8, 15, and 22,
- Rituximab 100 mg IV on days 1, 8, 15, and 22.

This regimen is distinguished by its early, simultaneous use of three agents with complementary mechanisms of action, rather than waiting for sequential failures. Dexamethasone serves as the corticosteroid backbone, while rituximab targets CD20-positive B cells responsible for autoantibody production, and cyclophosphamide provides broad cytotoxic suppression of immune activation. Originally developed and validated by our group through a multicenter analysis for the treatment of diverse forms of AHA, the CyDRi protocol has demonstrated exceptionally high rates of complete remission (∼97%) and an overall survival exceeding 90%, including in elderly patients and those with severe bleeding [38]. These outcomes surpass those typically achieved with traditional sequential immunosuppressive approaches, supporting the protocol’s utility as a first-line therapeutic option [38, 39]. This regimen is distinctive for its simultaneous use of all three agents from the outset, rather than the traditional stepwise escalation approach [38]. Key features that set it apart include the selection of dexamethasone as the steroid backbone, the coordinated timing of each drug’s administration to maximize synergistic immunosuppressive effects, and its successful application in both treatment-naïve and refractory cases of AHA [38]. In our recent study the CyDRi protocol has demonstrated a low incidence of adverse events [38]. Our postpartum patient (see below) also tolerated the regimen well, achieved complete remission within 6 weeks, and avoided infectious or cytopenic complications. The effectiveness of this approach in a young postpartum woman expands its applicability beyond elderly idiopathic AHA patients and supports its use as a first-line protocol in high-risk bleeding settings.

### Clinical illustration: postpartum AHA successfully treated with the CyDRi protocol

To illustrate the clinical features, diagnostic considerations, and therapeutic principles discussed above, we summarize the course of a young woman diagnosed and treated for postpartum AHA at our institution. The case is based on a retrospective review of medical records, laboratory data, and imaging studies. All diagnostic procedures, including coagulation assays and Bethesda inhibitor testing, were performed using standard protocols [3, 38], and the patient provided written informed consent for publication. Ethical approval was obtained from the South Pest Central Hospital/National Institute of Hematology and Infectious Diseases.

#### Initial clinical course

A 29-year-old woman with no previous bleeding history underwent an uncomplicated spontaneous vaginal delivery in December 2022. One week postpartum, she experienced persistent uterine bleeding requiring intramuscular oxytocin and 3 weeks of oral Ergometrine. Follow-up gynecological examination at 6 weeks was normal.

In January 2023, she sustained a blunt trauma to her left forearm. Within a week, she developed compartment syndrome requiring emergency fasciotomy due to venous bleeding and circulatory compromise in the distal forearm. Despite transfusions (6 units RBC, 2 units FFP), the surgical wound continued to ooze, prompting referral to our hematology unit.

#### Diagnostic evaluation

Upon admission to our department, the patient’s initial laboratory tests revealed a significantly reduced hemoglobin level of 82 g/L, consistent with ongoing blood loss. Routine coagulation screening demonstrated a markedly prolonged activated partial thromboplastin time (aPTT) of 74.3 s (reference range: 28–40 s), while prothrombin time and thrombin time remained within normal limits, suggesting an isolated defect in the intrinsic pathway of coagulation.

To further investigate the cause of the aPTT prolongation, we performed mixing studies. When patient plasma was combined in a 1:1 ratio with normal pooled plasma, the aPTT remained significantly prolonged after both immediate and 2-h incubation at 37 °C (95.2 s post-incubation), failing to normalize. This lack of correction strongly suggested the presence of an inhibitory antibody rather than a factor deficiency.

Subsequent specific coagulation factor assays confirmed an extremely low factor VIII activity of 1% (normal reference: 50%–150%), and a high-titer FVIII inhibitor was detected using the Bethesda assay, with a titer of 30.1 Bethesda Units (BU)/mL. Testing for lupus anticoagulant (LA), which may also cause prolonged aPTT, was negative, thereby excluding antiphospholipid syndrome and reinforcing the diagnosis of acquired factor VIII inhibitor.

Taken together — the postpartum timing, spontaneous and disproportionate bleeding, isolated non-correctable aPTT prolongation, severely depressed FVIII activity, and the presence of a high-titer inhibitor — confirmed the diagnosis of postpartum AHA.

#### Hemostatic and immunosuppressive management

Following the diagnosis of postpartum AHA, our clinical priorities were twofold: 1) immediate control of the life-threatening bleeding, and 2) eradication of the FVIII inhibitor through targeted immunosuppression.

Given the severity of the patient’s hemorrhage—manifesting as persistent oozing from the fasciotomy wound and rapidly declining hemoglobin despite transfusions—we initiated bypassing therapy with recombinant activated factor VII (rFVIIa, eptacog alfa) at a starting dose of 90 μg/kg administered every 3 hours. Despite this, bleeding from the surgical site persisted, necessitating further transfusion support; the patient ultimately received a total of 12 units of red blood cell concentrates during her hospitalization.

On day five of admission, the patient experienced a sudden increase in local bleeding volume, prompting urgent imaging to rule out arterial involvement. CT angiography of the left forearm revealed active contrast extravasation, consistent with arterial bleeding. The arterial hemorrhage identified on CT angiography was most likely related to trauma-associated vascular injury exacerbated by the underlying coagulopathy, rather than a primary surgical complication or pre-existing vascular abnormality. Vascular surgery was consulted, and the bleeding vessel was successfully ligated during an emergency surgical intervention. Postoperatively, bleeding markedly decreased, and transfusion needs gradually declined.

**FIGURE 1:**
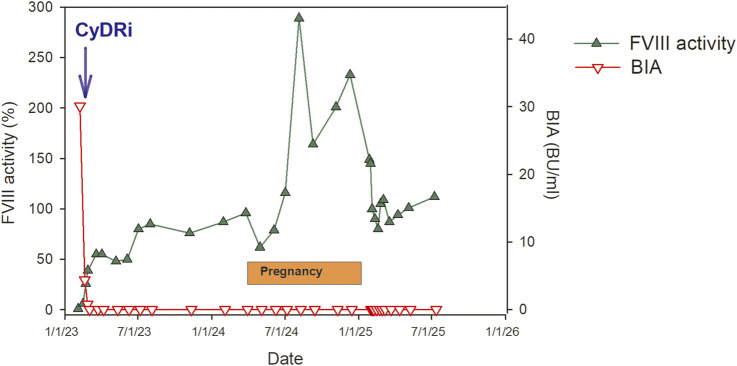
Long-term dynamics of factor VIII (FVIII) activity and inhibitor levels during CyDRi treatment and follow-up in postpartum acquired hemophilia A. This figure illustrates the hematologic response to the CyDRi immunosuppressive protocol (cyclophosphamide, dexamethasone, and rituximab) in a patient with postpartum acquired hemophilia A. The green curve represents FVIII activity (%), while the red curve denotes FVIII inhibitor titers measured using the Bethesda inhibitor assay (BIA; Bethesda Units/mL) over time. Key clinical events—including initiation of CyDRi therapy, subsequent pregnancy, and delivery—are indicated along the timeline. The figure demonstrates the rapid decline in inhibitor levels accompanied by progressive recovery of FVIII activity, culminating in complete remission within approximately 6 weeks, followed by sustained normalization during long-term follow-up. These dynamics highlight the efficacy and durability of CyDRi-based immunosuppression in achieving rapid inhibitor eradication and maintaining stable hemostatic function.

Simultaneously with hemostatic therapy, we initiated immunosuppressive treatment using the CyDRi protocol [3, 38] (Figure 1). Pain management during this period was also a clinical priority. The patient experienced significant postoperative discomfort at the fasciotomy site. Initially, she required intravenous morphine delivered via patient-controlled analgesia (PCA). However, due to adverse effects including nausea and somnolence, analgesia was transitioned to a transdermal fentanyl patch, which provided effective symptom control with improved tolerability.

Despite the use of rFVIIa, minor oozing from the wound persisted during the early immunosuppressive phase, and the dosing interval of rFVIIa was temporarily shortened to every 2 hours. As inhibitor titers began to fall and FVIII activity increased, the need for bypassing agents declined. After 19 days of rFVIIa therapy, the drug was safely discontinued.

By the third week of therapy, laboratory monitoring revealed rising FVIII activity and a steady decrease in inhibitor titer, indicating successful immune modulation. No treatment-related infections or neutropenic complications occurred during hospitalization. The patient was discharged in stable condition and entered outpatient follow-up.

#### Follow-up and pregnancy outcome

Following discharge, the patient was enrolled in a structured outpatient follow-up program with the hematology team. Her recovery was closely monitored with regular assessments of her coagulation profile, including FVIII activity and inhibitor titers.

By March 2023, approximately 6 weeks after completion of the CyDRi protocol, the patient achieved complete clinical and laboratory remission. Factor VIII activity had returned to normal range, and inhibitor levels were undetectable. She remained entirely asymptomatic, and no further bleeding episodes were reported.

In April 2023, she underwent elective plastic reconstructive surgery to correct the residual soft tissue defect from the prior fasciotomy on her left forearm. Remarkably, the procedure was performed without the use of bypassing agents or any special perioperative hemostatic support. There were no hemorrhagic complications, and wound healing proceeded uneventfully, further confirming durable remission of her acquired hemophilia A.

A particularly notable aspect of this case is what followed: in June 2024, the patient became pregnant. Given the known association between pregnancy/postpartum immune shifts and the development of AHA, her new pregnancy was considered high risk from a hematologic standpoint. Therefore, a multidisciplinary management plan was implemented, involving hematologists and obstetricians.

Throughout the pregnancy, FVIII activity and aPTT were monitored monthly. At no point during gestation did her laboratory parameters indicate inhibitor recurrence. FVIII levels remained within the normal range, and aPTT values were consistently unremarkable. The pregnancy progressed without complications, and no clinical signs of bleeding, bruising, or other hemostatic disturbances emerged.

In January 2025, she delivered a healthy male infant via spontaneous vaginal delivery at term. The peripartum period was closely supervised with continuous coagulation monitoring. No bleeding complications occurred during labor or delivery. Postpartum observation was prolonged to detect any early signs of relapse; however, no laboratory or clinical abnormalities were observed. The postpartum course remained uneventful, and both mother and newborn were discharged in good health.

This favorable obstetric outcome, following successful treatment of postpartum AHA, is rare and significant. It suggests that with effective immune eradication and vigilant follow-up, subsequent pregnancies may proceed safely, even in patients with a history of high-titer FVIII inhibitors.

#### Clinical implications and conclusions

The patient described here exemplified the typical presentation of postpartum AHA, with delayed-onset, life-threatening bleeding and no prior history of bleeding disorders. The clinical progression highlights a hallmark of AHA: the disproportion between the bleeding severity and apparent local injury, which should always prompt consideration of an acquired inhibitor.

This case underscores several critical principles for the diagnosis, treatment, and long-term management of postpartum AHA. In postpartum women presenting with atypical, delayed, or severe bleeding—particularly when disproportionate to obstetric findings—clinicians must consider acquired coagulopathies such as AHA early in the diagnostic process. Prompt access to coagulation studies, especially aPTT, mixing tests, FVIII assays, and inhibitor titers, is essential for rapid diagnosis and intervention.

The combination of bypassing therapy (rFVIIa) and a structured, frontline immunosuppressive regimen using the CyDRi protocol led to rapid clinical stabilization and complete remission. Notably, she tolerated the CyDRi regimen well and experienced no infectious or hematologic complications, supporting its applicability in postpartum patients beyond its established efficacy in elderly or idiopathic AHA cases.

Importantly, this case provides rare and encouraging evidence that future pregnancies may proceed safely after successful treatment. The patient’s subsequent gestation and spontaneous vaginal delivery were entirely uneventful, with no signs of inhibitor recurrence. While data on relapse risk in future pregnancies remain sparse, this case suggests that durable immune tolerance can be achieved and maintained, provided that patients undergo careful monitoring and multidisciplinary planning.

Taken together, this case reinforces the importance of early recognition, coordinated care, and the use of effective, low-toxicity treatment strategies in postpartum AHA. It also offers a hopeful outlook for women of childbearing age diagnosed with this rare condition, indicating that with appropriate management, complete recovery and healthy future pregnancies are possible.

## Reproductive considerations and safety of immunosuppressants

A pivotal and underexplored question in postpartum AHA is whether subsequent pregnancies are safe. Recurrence of AHA during a second pregnancy is biologically plausible given the role of pregnancy-related immune shifts in pathogenesis. However, data are sparse and largely anecdotal. Our case adds valuable prospective data: the patient conceived approximately 1 year after remission and experienced an entirely uneventful pregnancy, with no laboratory evidence of FVIII inhibitor recurrence and a spontaneous vaginal delivery free of bleeding complications. This suggests that complete eradication of the inhibitor and durable immune tolerance can be re-established, allowing safe future childbearing. Nonetheless, we recommend preconception counseling and meticulous peripartum surveillance in such cases.

The safety of immunosuppressive therapy used in the management of AHA is a critical consideration in women of reproductive age, particularly during the antepartum and preconception periods. Among the agents used in the CyDRi protocol, cyclophosphamide carries a known risk of premature ovarian insufficiency and infertility, which is dose- and age-dependent. The total cumulative dose appears to be the most significant determinant of long-term reproductive toxicity. Evidence suggests that a longer interval between the final dose of cyclophosphamide and conception is associated with improved pregnancy outcomes, with a recommended washout period of at least 3 months prior to attempting conception [40, 41]. However, for women who receive higher cumulative doses or who are approaching advanced reproductive age, a longer interval may be prudent.

Rituximab, the anti-CD20 monoclonal antibody component of the CyDRi protocol, is known to cross the placenta—primarily after the first trimester—and has been associated with transient neonatal B-cell depletion, hypogammaglobulinemia, and cytopenias. Although these effects are typically self-limited, current guidelines recommend discontinuing rituximab prior to conception, and advise against its routine use during pregnancy unless clearly indicated [41, 42]. In the context of our case, where immunosuppressive treatment was administered postpartum, the CyDRi protocol was not only effective but also compatible with future reproductive safety, given the relatively low cumulative cyclophosphamide dose and the extended washout period prior to conception. This highlights the importance of individualized reproductive planning and multidisciplinary coordination when initiating immunosuppressive therapy in young women with AHA. In postpartum cases, such as the one described here, the timing of immunosuppression relative to delivery provides a unique advantage for minimizing reproductive risk, but careful preconception counseling and personalized follow-up remain important.

## Conclusions and future directions

This review underscores the importance of maintaining a high index of suspicion for AHA in any postpartum patient with unexplained or disproportionate bleeding and an isolated prolonged aPTT. Our illustrative case highlights the value of rapid diagnostic work-up and the early initiation of both hemostatic and immunosuppressive therapy. The successful application of the CyDRi protocol in this setting demonstrates its efficacy and tolerability, even in patients presenting with severe hemorrhage. Importantly, this case also offers rare insight into the long-term reproductive outcome following postpartum AHA. The patient’s subsequent healthy pregnancy and uneventful delivery suggest that, with appropriate follow-up and multidisciplinary care, future gestations can proceed safely.

Despite these encouraging observations, postpartum AHA remains poorly understood. Further research is needed to clarify its immunogenetic underpinnings, optimal therapeutic strategies, and implications for future fertility and maternal health. Longitudinal data from multicenter registries and prospective studies are essential to better define long-term outcomes and to inform evidence-based guidelines for managing this rare condition.
